# Intratumoral CXCR4^hi^ neutrophils display ferroptotic and immunosuppressive signatures in hepatoblastoma

**DOI:** 10.3389/fimmu.2024.1363454

**Published:** 2024-02-29

**Authors:** Zhengjing Lu, Xiaolin Wang, Jun Feng, Wenjia Chai, Wei Wang, Qixin Wang, Shen Yang, Wei Yang, Yan Su, Wenjun Mou, Yun Peng, Huanmin Wang, Jingang Gui

**Affiliations:** ^1^ Laboratory of Tumor Immunology, Beijing Pediatric Research Institute, Beijing Children’s Hospital, Capital Medical University, National Center for Children’s Health, Beijing, China; ^2^ Key Laboratory of Major Diseases in Children, Ministry of Education, Beijing Pediatric Research Institute, Beijing Children’s Hospital, Capital Medical University, National Center for Children’s Health, Beijing, China; ^3^ Department of Surgical Oncology, Beijing Children’s Hospital, Capital Medical University, National Center for Children’s Health, Beijing, China; ^4^ Beijing Key Laboratory of Pediatric Hematology Oncology, National Key Discipline of Pediatrics, Ministry of Education, Key Laboratory of Major Diseases in Children, Hematology Oncology Center, Beijing Children’s Hospital, Capital Medical University, National Center for Children’s Health, Beijing, China

**Keywords:** neutrophil, ferroptosis, immunosuppression, hepatoblastoma, CXCR4

## Abstract

Pediatric hepatoblastoma (HB) is the most common primary liver malignancy in infants and children. With great diversity and plasticity, tumor-infiltrating neutrophils were one of the most determining factors for poor prognosis in many malignant tumors. In this study, through bulk RNA sequencing for sorted blood and tumor-infiltrated neutrophils and comparison of neutrophils in tumor and para-tumor tissue by single-cell sequencing, we found that intratumoral neutrophils were composed of heterogenous functional populations at different development stages. Our study showed that terminally differentiated neutrophils with active ferroptosis prevailed in tumor tissue, whereas, in para-tumor, pre-fate naïve neutrophils were dominant and ferroptotic neutrophils dispersed in a broad spectrum of cell maturation. Gene profiling and *in vitro* T-cell coculture experiment confirmed that one of main functional intratumoral neutrophils was mainly immunosuppressive, which relied on the activation of ferroptosis. Combining the bulk RNA-seq, scRNA-seq data, and immunochemistry staining of tumor samples, CXCL12/CXCR4 chemotaxis pathway was suggested to mediate the migration of neutrophils in tumors as CXCR4 highly expressed by intratumoral neutrophils and its ligand CXCL12 expressed much higher level in tumor than that in para-tumor. Moreover, our study pinpointed that infiltrated CXCR4^hi^ neutrophils, regardless of their differential distribution of cell maturation status in HB tumor and para-tumor regions, were the genuine perpetrators for immune suppression. Our data characterized the ferroptosis-dependent immunosuppression energized by intratumoral CXCR4 expression neutrophils and suggest a potential cell target for cancer immunotherapies.

## 1 Introduction

Hepatoblastoma (HB) is the most common primary liver malignancy in children. Most are diagnosed in children younger than 3 years of age (1, 2). Tumor stage, alpha-fetoprotein, multifocality, histological type, type of radical surgery, metastasis, and adjuvant therapy such as chemoradiation are major determinants for the HB prognosis (3). Because of tumor heterogeneity and immunosuppressive microenvironment of HB, patients incurred poor outcomes such as metastases and tumor relapses, regardless of the improved overall survival (OS) rate benefited from surgical resection in combination with neoadjuvant chemotherapy (4–6). To be noted, as one of the most ominous predictors for tumor progression, how intratumoral neutrophils engage themselves to reinforce immunosuppressive microenvironment remains a mystery, particularly in pediatric tumor such as HB.

Within the tumor microenvironment (TME), neutrophils are functionally perturbed and show a high level of plasticity in response to signals that drive their polarization and activation, indicating that they are potential therapeutic targets for cancer treatment (7). Neutrophils have been shown to impair T-cell response through PD-L1–mediated contact-dependent mechanisms (8). Moreover, neutrophils possess versatile means in promoting tumor progression, including generating neutrophil extracellular traps (NETs) (9), reactive oxygen species (ROS) (10), arginase 1 (11, 12), and myeloperoxidase (13). Previous study identified a neutrophil spectrum in liver cancer and decoded that CCL4^+^ neutrophils were able to recruit macrophages and that PD-L1^+^ neutrophils suppressed T-cell cytotoxicity. Recently, it has been demonstrated that ferroptotic neutrophils could release diverse lipid mediators, such as prostaglandin E2 to inhibit anti-tumor functions of innate and adaptive immune cells (14–18). The fact that inhibition of ferroptosis together with immune checkpoint blockade abrogated the immunosuppressive activity of neutrophils and prevented tumor progression implies that neutrophil ferroptosis is a potential target for cancer therapy (14). Moreover, existing evidence has shown that, despite of the short lifespan, neutrophils are guided by tissue-derived chemokine signals (for instance, CXCL12/CXCR4 axis) to specific sites where they adopted properties tailored to the needs of tissue (19). Neutrophil reprogramming in tissues occurs in CXCL12-rich niches with upregulated CXCR4. Compared with CXCR4^lo^ neutrophils, CXCR4^hi^ neutrophils have enhanced NET formation, phagocytic function, neutrophil degranulation, and overexpression of pro-inflammatory cytokines and chemokines *in vitro* (20).

Despite several attempts to explore the immune cell function in HB, the effect of ferroptosis on immune cells, especially neutrophils, remains poorly understood (21). Here, we provided evidence that HB tissue–infiltrated neutrophils were immunosuppressive and had a higher susceptibility to ferroptosis. A group of intratumoral CXCR4^hi^ neutrophils was further demonstrated to have higher tendency to undergo ferroptosis and express immuno-inhibitory molecules to suppress T-cell cytotoxicity to HB tumor cells.

## 2 Materials and methods

### 2.1 Human subjects

Clinical data from 127 patients diagnosed with HB based on histology, cytology, and typical imaging evidence in the Capital Medical University Affiliated Beijing Children Hospital between September 2016 and December 2022 were retrospectively reviewed. All of the patients were treated in accordance with the guidelines from the Children’s Hepatic Tumors International Collaboration–Hepatoblastoma Stratification (CHIC-HS) system. Patients who had fever; allergy or infection caused by any pathogen; myelosuppression or liver insufficiency within 1 week before operation; and thymus-related diseases, spleen-related diseases, blood system diseases, autoimmune diseases, or immune deficiencies were excluded.

Five tumor samples from 18 enrolled patients with HB in Beijing Children Hospital were used for single-cell RNA sequencing (scRNA-seq) and others for neutrophil functional analysis. The clinical data of 18 patients were listed in **Supplementary Table 1**. In addition, blood samples from age-matched healthy donors were collected for *in vitro* immune functional assays. The study protocol was approved by the ethics committee of Capital Medical University affiliated Beijing Children Hospital. Parents of all patients consented to attend this cohort study and signed a written informed consent.

### 2.2 Sample collection, dissociation, and cell purification

Peripheral blood samples were collected using ethylenediamine tetraacetic acid (EDTA) anticoagulant tubes prior to surgery and processed within 15 min after collection. Blood neutrophils were harvested after lyses of red blood cells by red blood cell lysis buffer (Solarbio, Beijing, China). Then, CD15^+^ neutrophils were sorted out using CD15-positive selection magnetic beads following the manufacturer’s protocol (Miltenyi Biotec, BergischGladbach, Germany). CD3^+^ T cells and CD8^+^ T cells were isolated by using anti-CD3 and anti-CD8 magnetic beads (Miltenyi Biotec).

Fresh tumors and para-tumors were cut into approximately 1-mm^3^ pieces in Dulbecco’s modified eagle medium (DMEM; Corning) and enzymatically digested using the magnetic activated cell sorting (MACS) tumor dissociation kit (Miltenyi Biotec) for 30 min at 37°C, according to the manufacturer’s instructions. Being filtered by 100-μm cell strainers (BD) in DMEM medium, the suspended cells were centrifuged at 400g for 5 min. Cell pellets were resuspended in sorting buffer (autoMACS^®^ rinsing solution supplemented with 5% bovine serum albumin (BSA)) after washing twice with phosphate buffered saline (PBS). Tumor- and para-tumor–infiltrated neutrophils were similarly isolated by CD15 magnetic beads (Miltenyi Biotec).

Throughout the dissociation and isolation procedure, cells were maintained on ice whenever possible. The viability (>90%) and purity (>95%) of sorted cells were determined by flow cytometry with an LSRFortessa X-20 flow cytometer (BD Biosciences, San Jose, CA, USA) and analyzed with FlowJo software (version 10).

### 2.3 Bulk RNA sequencing and bioinformatic analysis

RNA-seq was performed in sorted CD15^+^ neutrophils from peripheral blood (HBPB, n = 4), tumor tissues (HBT, n = 5), and para-tumors (HBPT, n = 3) of patients with HB. Total RNA was extracted from neutrophils using TRIzol reagent (Invitrogen, Carlsbad, CA, USA) and stored at −80°C for subsequent RNA-seq (OEbiotech, China). RNA purity and quantification were evaluated using the NanoDrop 2000 spectrophotometer (Thermo Fisher Scientific Inc., Waltham, MA, USA). RNA integrity was assessed using the Agilent 2100 Bioanalyzer (Agilent Technologies, Santa Clara, CA, USA). Then, the libraries were constructed using the VAHTS Universal V6 RNA-seq Library Prep Kit according to the manufacturer’s instructions. The library was quality-checked and sequenced on the NovaSeq 6000 platform (Illumina). The quality of sequencing reads was evaluated using FastQC. Adaptor sequences and bases with low-quality score were trimmed using trimmomatic (v.0.36). The image data measured by the high-throughput sequencer are converted into sequence data (reads) by CASAVA base recognition. Reference genome and gene model annotation files were downloaded from genome website directly. The fragments per kilobase of exon per million mapped reads values and gene count values were computed using RNA-Seq by Expectation Maximization (RSEM) (v.1.3.1), and differentially expressed genes (DEGs) were analyzed using the DESeq2 (v.1.24) R package. Gene expression level with log_2_ fold change ≧ 1 or ≦ −1 and an adjusted q-value < 0.05 were defined as DEGs in further analysis. The bioinformatic analysis was performed according to the instructions of the databases and online platforms (https://cloud.oebiotech.cn/task/).

### 2.4 Single-cell RNA sequencing, unsupervised clustering, and cell-type annotation

scRNA-seq was performed on the single-cell suspensions with viability >70%. DNBelab C Series High-throughput Single-Cell Library (MGI, Shenzhen, China) was utilized for scRNA-seq library preparation. Whole-transcriptome libraries were prepared according to the BD Rhapsody single-cell whole-transcriptome amplification workflow. Sequencing libraries were then quantified by using the Qubit ssDNA Assay Kit (Thermo Fisher Scientific) on a Bioanalyzer 2100 (Agilent Technologies) and the Qubit High Sensitivity DNA assay (Thermo Fisher Scientific). Pair-end sequencing was done using a DNBSEQ-T7 (MGI, Shenzhen, China) sequencing platform. Cell clustering was conducted by the Seurat (v4.3) package in R software (R version 4.0.4). Genes expressed in less than three cells were filtered out, and cells with fewer than 200 genes were excluded. The quality of cells was assessed on the basis of two metrics: (1) the number of detected genes was above 200 and below 5,000 and (2) the percentage of mitochondrial genes was below 25. The 21 libraries were then integrated with the *Merge* and *harmony* functions, and the batch effects were checked if the cells were separately distributed with the *DimPlot* function. Then, the integrated data were scaled to calculate the principal component (PC) analysis. The first 50 PCs were used to construct the shared nearest neighbor network, and the graph-based clustering method Louvain algorithm was used to identify the cell clusters with a resolution of 0.9 across highly variable genes (4,000). Finally, uniform manifold approximation and projection (UMAP) was used to visualize the clustering results in two-dimensional space. To annotate each cluster as a specific cell type, we selected known classic markers of immune cells, fibroblasts, hepatocytes, and hepatoblasts (22–25).

### 2.5 scRNA-seq analysis strategy

To measure the ferroptotic status of different cell types, we scored cells using *AddModuleScore* with a set of characteristic genes involved in ferroptosis (14). We applied “monocle” to order neutrophils in pseudo-time to indicate their developmental trajectories. To visualize the ordered cells in the trajectory, we used the plot_cell_trajectory function to plot the minimum spanning tree on the cells. The starting point of the pseudo-time trajectory was determined on the basis of preliminary understanding of the cell populations used in the analysis. *BEAM* was used for further analysis of neutrophil signatures in different fate branches. We used *CellChat* to infer cell–cell interaction between different cell types. This method infers the potential interaction strength between two cell subsets based on gene expression levels. Dot plots were then plotted using these files to illustrate only the significant ligand-receptor interactions.

### 2.6 Transmission electron microscopy

Neutrophils were fixed by the electron microscope fixative solution at room temperature (RT) for 1 h and maintained at 4°C until use. Fixed cells were dehydrated and epoxy-embedded, followed by incubation at 60°C for 48 h. Finally, ultrathin (60 nm) slices were collected on grids and then stained with 1% uranyl acetate and 0.4% lead citrate. Images were obtained using a Hitachi 7700 transmission electron microscopy (Hitachi, Tokyo, Japan).

### 2.7 Flow cytometry

For neutrophil phenotyping, cells were stained using the following antibodies: anti-CD45, anti-CD15, anti-CD54, anti–PD-L1, anti-CD71, anti-CXCR1, anti-CXCR2, anti-CXCR4, and anti-CD62L. To assess T-cell activation, cells were stained with anti-CD3, anti-CD4, anti-CD8, anti-CD25, and anti-CD69. All antibodies were purchased from BioLegend (San Diego, CA, USA). Sample acquisition was done with an LSRFortessa X-20 flow cytometer, and data were processed by FlowJo software (version 10). Gating of interest was properly placed around populations of cells with common characteristics.

### 2.8 Lipid ROS assay

The relative lipid ROS level in isolated neutrophils was assessed using C11-BODIPY probe (Dojindo Molecular Technology, Japan). Being treated with 10 μM C11-BODIPY for 30 min, cells were harvested, washed twice with Hank’s balanced salt solution, resuspended, and then subjected to flow cytometric analysis by an LSRFortessa X-20 flow cytometer.

### 2.9 Detection of intracellular Fe^2+^

The Fe^2+^ probe FerroOrange (1 μM, Dojindo Molecular Technology) was used to measure the intracellular free Fe^2+^ ion according to the manufacturer’s instruction. The fluorescence intensities of the isolated neutrophils were quantified on an LSRFortessa X-20 flow cytometer. Data were analyzed with FlowJo v.10 software.

### 2.10 Preparation of TTCS and PTCS and supernatant-conditioned neutrophils

Tumor tissue culture supernatants (TTCSs) or para-tumor tissue culture supernatants (PTCSs) were collected from culture of single–tumor cell suspension at a concentration of 1 × 10^6^ cells/mL for 48 h.

To generate supernatant-conditioned neutrophils, isolated neutrophils were suspended in RPMI 1640 medium at 1 × 10^6^ cells/mL and stimulated with 50% TTCSs or PTCSs at 37°C for 6 h to 16 h. Neutrophils cultured with RPMI 1640 medium without stimulation were used as controls.

### 2.11 *In vitro* neutrophil–T-cell coculture

The isolated CD3^+^ T cells were stimulated with anti-CD3 (5 μg/mL) and anti-CD28 (5 μg/mL) antibodies (BioLegend). TTCS-conditioned or control medium-cultured neutrophils was added to a round-bottomed 96-well plate, in the presence of anti-human PD-L1 (10 μg/mL) neutralizing antibody. In some cultures, ferroptosis inhibitor liproxstain-1 (LPX1; 50 μM) or ferroptosis inducer RSL3 (10 μM) was added taking DMSO as a reagent for control experiments. After 48 h of incubation, cells were stained with T-cell activation markers CD25 and CD69 (BioLegend), and cell events were recorded by an LSRFortessa X-20 flow cytometer and analyzed by FlowJo v.10 software.

### 2.12 Real-time cellular analysis cytotoxicity assay

HuH6 cells were seeded into 16-well E-plates (Agilent Technologies) at 1 ×10^4^ cells per well, and monitored overnight with the xCELLigence real-time cell analyzer (Agilent Technologies, CA, USA) according to the manufacture’s instruction. xCELLigence real-time cellular analysis (RTCA) measured cellular adhesion through electrical impedance, which was then converted into Cell Index. Microbeads isolated CD8^+^ T cells from abovementioned neutrophil–T-cell coculture were then added into HuH6 culture when the cell index reached a plateau at a 10:1 effector-to-target ratio. The cells in the E-plates were monitored for another 72 h, and the impedance changes were plotted over time with the RTCA system.

### 2.13 Enzyme linked immunosorbent assay (ELISA)

The expression of CXCL12 in HuH6 cultured supernatants (HuH6-CS), TTCS, and PTCS was determined using the human CXCL12/SDF-1 ELISA Kit (MultiSciences Biotech, Hangzhou, China) in accordance with the manufacturer’s recommendations. In brief, samples or standards were added to the wells and incubated for 120 min at room temperature. After washing, a working detector was added to each well, followed by the addition of the substrate solution. The reaction was stopped, and the absorbance was read at 450 nm.

### 2.14 Chemotaxis of neutrophil

Chemotaxis of neutrophil was evaluated using a 96-well Boyden chamber (Corning, Kennebunk, ME, USA) with 3-μm–pore size polyvinylpyrrolidone-free polycarbonate membranes (36154611). In brief, 200 μL of RPMI 1640 medium alone (as control) or supplemented with 10% TTCSs or PTCSs, were added in the bottom chamber. Subsequently, 2 × 10^5^ neutrophils suspended in 75 μL of RPMI 1640 medium were added to the top chamber and allowed to migrate for 16 h at 37°C. Migrated cells in the bottom well were eventually counted by a flow cytometer using flow cytometric Precision Counting Beads (BioLegend, San Diego, CA, USA).

### 2.15 Immunohistochemistry staining

Formalin-fixed and paraffin-embedded tissues being sectioned to 4 μm were used for histology evaluation of HB tumors. Tissue sections were stained with anti-CXCL12 (1:500, Proteintech, Rosemont, IL, USA) antibody overnight at 4°C, followed by nuclear counterstaining with Hematoxylin Gill III (1.05174.0500, Merck). The slides were then incubated with horseradish peroxidase (HRP)-conjugated secondary antibody for 10 min at 37°C. Finally, the sections were visualized by diaminobenzidine solution and counterstained with hematoxylin. The stained slides were scanned at a resolution of ×20 using the Vectra Polaris Slide Scanner (Akoya Biosciences, Marlborough, MA, USA) and viewed by the CaseViewer software (V2.3.0).

Histochemistry score was used for semi-quantitative analysis of the CXCL12 protein expression level, and it was evaluated using the following formula: CXCL12-score = the staining intensity × the rate of positive cells (26, 27). The intensity of positive cells was graded as 0 (negative, unstained), 1 (weak), 2 (moderate), and 3 (strong). The score of positive cells was recorded as follows: 0, less than 5% CXCL12-positive cell infiltration; 1, 5%–25% CXCL12-positive cells; 2, 25%–50% CXCL12-positive cells; 3, 50%–75% CXCL12-positive cells; and 4, more than 75% CXCL12-positive cell infiltration (28).

### 2.16 Quantitative real-time PCR

Total RNA from neutrophils was purified using the Direct-zol RNA Miniprep (ZYMO research, Orange, CA, USA) following the manufacturer’s instruction. RNA concentration was measured by a NanoDrop ND-8000 (Thermo Fisher Scientific Inc., Waltham, MA, USA). Reverse transcription was performed according to standard protocols using a RevertAid First-Strand cDNA Synthesis Kit (Thermo Fisher Scientific Inc., Waltham, MA, USA). Quantitative PCR was performed using the SYBR Green PCR Master Mix (TIANGEN, Beijing, China), and the fluorescence was recorded by a QuantStudio 6 flex real-time PCR system (Applied Biosystems, Foster City, CA, USA). Relative expression was calculated by the 2^−ΔΔCt^ method with glyceraldehyde-3-phosphate dehydrogenase (GAPDH) as the endogenous housekeeping gene control. The primer sequences referred to the previous study (14).

### 2.17 Statistics

Statistical analysis was conducted by using GraphPad Prism 9.0 software (GraphPad Software, Inc., La Jolla, CA, USA). All experiments were repeated at least three times. Only representative data are shown. All results were reported as mean ± standard error of mean (SEM). Comparison of the variables between groups was made by Student’s *t*-test. *p*-value < 0.05 indicated a statistically significant difference: * *p* < 0.05, ** *p* < 0.01, *** *p* < 0.001, and **** *p* < 0.0001.

## 3 Results

### 3.1 Augmentation of neutrophils and increased NLR in blood of patients with HB was associated with poor prognosis

**Figure 1:**
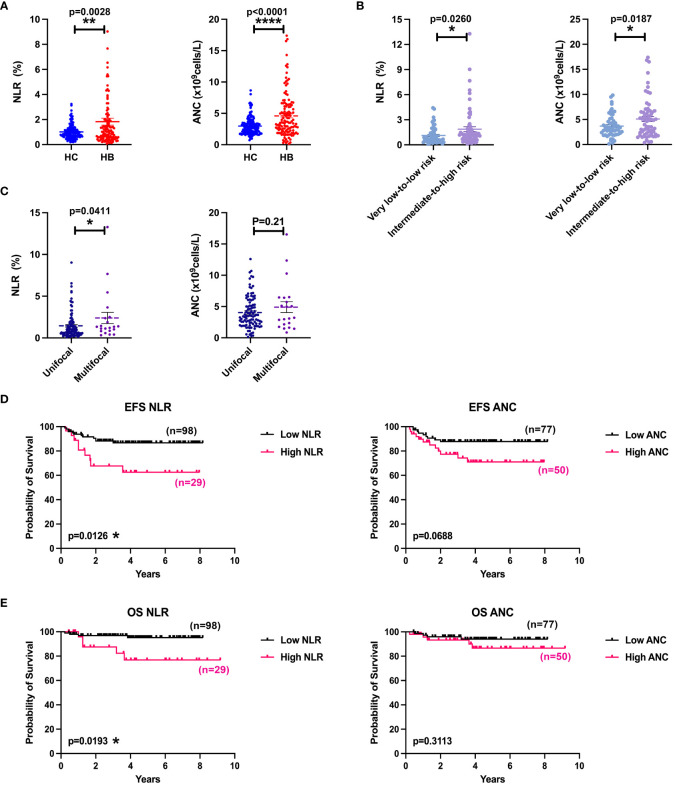
Increased NLR and ANC predicted poor survival. **(A)** Comparison of NLR or ANC values between HCs (n = 160) and patients with HB (n = 127). **(B)** Comparison of NLR or ANC values between low-risk group (n = 61) and medium-to-high-risk group (n = 66). **(C)** Comparison of NLR or ANC values between unifocal (n = 106) and multifocal (n = 21) HB groups. **(D)** EFS curves of patients with HB according to NLR (cutoff = 2.00) and ANC (cutoff = 4.65). **(E)** OS curves of patients with HB according to NLR (cutoff = 2.00) and ANC (cutoff = 4.65). Results for all measurements were mean ± SEM. *p < 0.05, **p < 0.01, and ****p < 0.0001.

It has been reported that elevated neutrophil-to-lymphocyte ratio (NLR) in peripheral blood of patients with HB predicts poor OS (29). To evaluate the potential role of neutrophils in HB, we compared NLR and absolute neutrophil count (ANC) in blood samples from 127 patients with HB with controls from 160 healthy donors (**Figure 1A**). As expected, patients with HB showed a remarkably higher NLR and ANC in peripheral blood than healthy controls (HCs). In children, the production of neutrophils changes along with age. We then normalized the ratios and absolute numbers by five age intervals according to reference intervals of blood cell analysis for children (WS/T779—2021) and confirmed the consistence in augmentation of NLR and ANC in patients with HB (**Supplementary Figure 1**). Patients were divided into the very low–to–low-risk group and the intermediate-to-high-risk group based on the risk stratification criteria of the CHIC-HS. Indeed, patient group in intermediate-to-high risk presented with significantly increased NLR and ANC in comparison with very low-to-low-risk patient group (**Figure 1B**). Because multifocality has been confirmed as an independent risk factor (30), we re-grouped the patients bearing multifocal tumors from those with unifocal tumors. Expectedly, NLR was consistently higher in patients with multifocal HB in spite of the equivalent of ANC (**Figure 1C**). Our analysis further revealed that blood NLR but not ANC presented with strong negative associations with OS and event-free survival (EFS) (**Figures 1D, E**). Together, these data implied that increased NLR is one of the key adverse prognostic factors.

### 3.2 Bulk RNA-seq of intratumoral neutrophils revealed a robust ferroptosis gene signature

**Figure 2:**
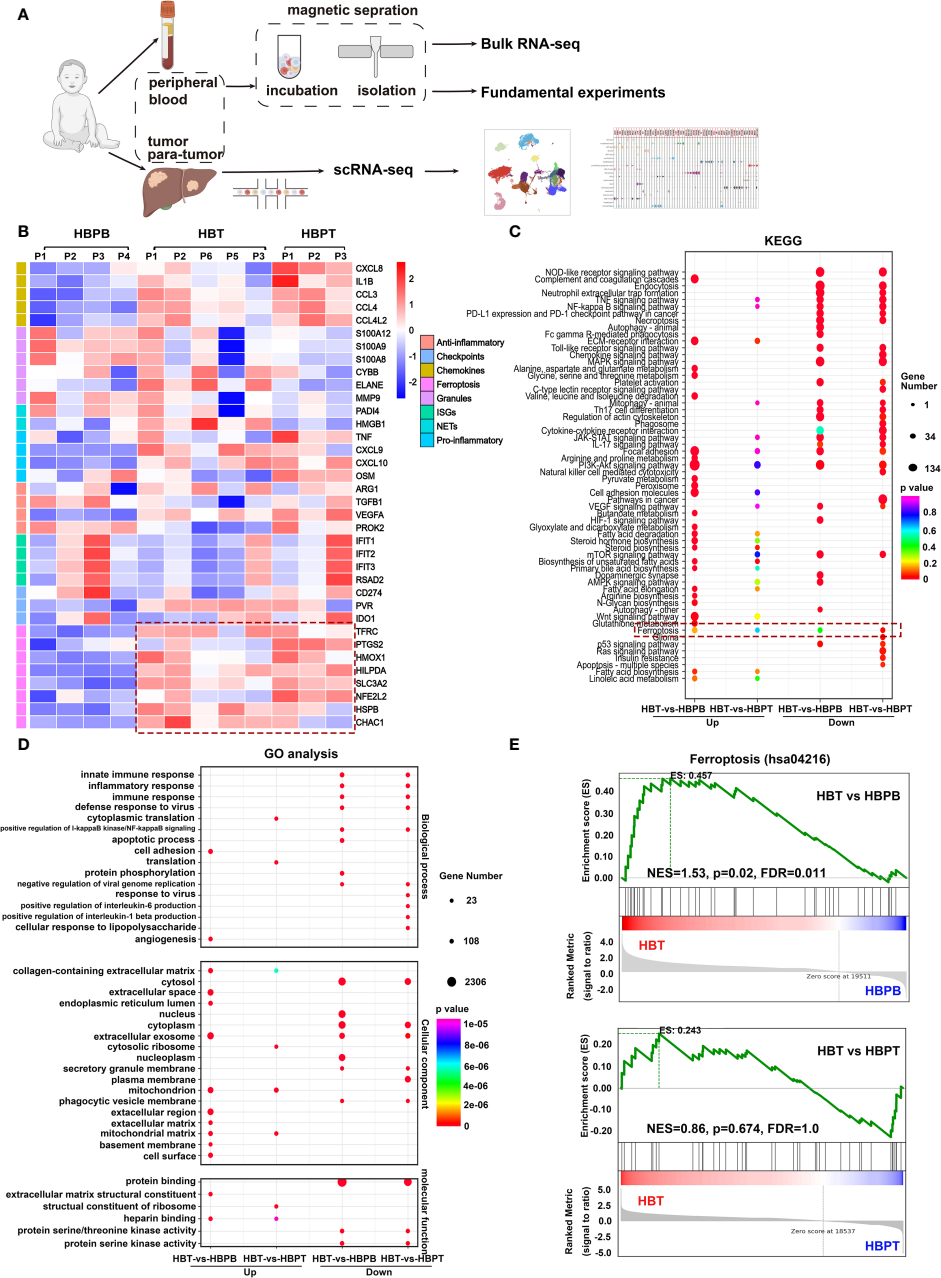
Immunoprofile and differentially expressed genes (DEGs) in HB neutrophils. **(A)** Flow chart of experimental protocol used in this study. **(B)** Heatmap of expression of neutrophil marker genes. NETs, neutrophil extracellular traps; ISGs, interferon-stimulated genes. **(C)** KEGG pathway enrichment analysis of DEGs. **(D)** GO enrichment analysis of DEGs. **(E)** Gene set enrichment analysis (GSEA) of ferroptosis pathway (hsa04216) in neutrophils.

Prompted by data supporting the prognostic significance of neutrophils, we investigated the gene expression profiles by bulk RNA-seq and scRNA-seq and performed experiments for validation (**Figure 2A**). As shown in **Supplementary Figure 2A**, following quality control and normalization, HBT, HBPT, and HBPB neutrophil exhibited clear demarcations of gene expression by PC analysis. A total of 5,425 upregulated and 3,846 downregulated DEGs were revealed between the HBT and HBPB groups, as well as a total of 2,018 upregulated and 2,295 downregulated DEGs for HBT versus HBPT groups (**Supplementary Figure 2B**). Further analysis of bulk RNA-seq data revealed appreciable different gene signatures between neutrophils in each respective group (**Figure 2B**). HBT and HBPT neutrophils exhibited a strong potential for chemokine and pro-inflammatory cytokine production. Furthermore, immune checkpoint molecules, such as poliovirus receptor and indoleamine 2,3-dioxygenase 1, were elevated in both HBT and HBPT neutrophils compared with those expressed in HBPB neutrophils. On the basis of the bulk RNA-seq from isolated neutrophils, it attracted our attention that both HBT and HBPT neutrophils exhibited robust ferroptosis gene signature, suggesting a distinct functional change occurred from peripheral blood niche to TME. Note that, compared with HBPT neutrophils, peptidyl arginine deiminase 4 (PADI4) and high mobility group box 1 (HMGB1) involved in NET formation, as well as cell activation–related genes, were enriched in HBT neutrophils, indicating a strong interaction between neutrophils and tumor cells. Kyoto Encyclopedia of Genes and Genomes (KEGG) pathway enrichment analysis and gene ontology (GO) functional annotation were performed and the 40 enriched pathways were shown (**Figures 2C, D**). In terms of KEGG analysis, pathways, including Phosphatidylinositol-3-kinase (PI3K)-Akt signaling pathway, extra cellular matrix (ECM)–receptor interaction, glycerolipid metabolism, cell adhesion molecules, biosynthesis of steroid hormone, unsaturated fatty acids, ferroptosis and its related glutathione metabolism, and peroxisome generation, were enriched in HBT neutrophils, whereas chemokine signaling pathway was enriched in HBPB neutrophils (**Figure 2C**). GO functional analysis showed enriched cell adhesion, collagen-containing extracellular matrix, mitochondrion, and mitochondrion matrix pathways in HBT neutrophils (**Figure 2D**). According to KEGG and GO analysis results, we found that HBT neutrophils showed deviated chemokine signaling and ferroptosis signatures, as well as ferroptosis-related lipid metabolism, lipid oxidation, and mitochondrial function signatures.

To ensure our findings, we analyzed the bulk RNA-seq data by gene set enrichment analysis (GSEA) so that genes related to particular biological states were considered as a whole. In consistent with analysis from directly comparing relative intensity of gene expression separately, gene set analysis revealed an enhanced ferroptosis (hsa04216) in HBT neutrophils compared with that in HBPB neutrophils, whereas there was a comparable ferroptosis score with no significant difference between HBT and HBPT neutrophils (**Figure 2E**).

### 3.3 Single-cell RNA sequencing indicated that majority of intratumoral neutrophils was at terminal fate state with high ferroptotic level

**Figure 3:**
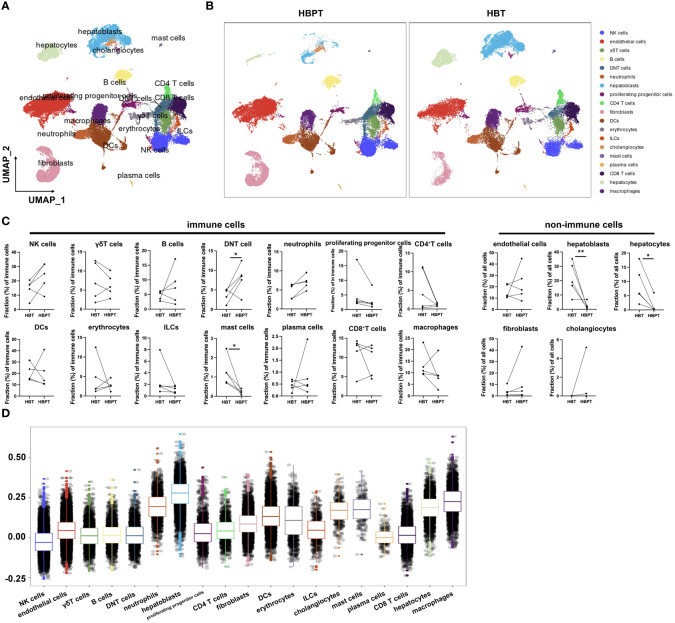
Single-cell profiling of patients with HB reveals distinctive tumor-infiltrated cell populations. **(A)** UMAP plots of all cells annotated by cell types according to canonical markers. **(B)** UMAP plots of distribution discrepancy of cell clusters between HBT and HBPT. **(C)** Dot plots showing cellular fractions of immune cells and non-immune cells in HBT (n = 5) and paired HBPT (n = 5). **(D)** Box plots of ferroptosis signature score in different cell types. Results for all measurements were mean ± SEM. Significant differences between groups were determined by *p < 0.05 and **p < 0.01.

To provide a deeper insight into neutrophil populations in HB TME, we performed scRNA-seq using HB tumor tissues (n = 5) and paired para-tumor tissues (n = 5). After initial stringent quality control, we obtained usable data from a total of 34,355 cells. As illustrated by UMAP in **Figure 3A**, 19 cell clusters including 14 types of immune cells and five types of non-immune cells were annotated based on the expression of corresponding canonical marker genes (**Figures 3A, B**; **Supplementary Figures 3**, **4**). Among them, hepatoblasts, hepatocytes, mast cells, and macrophages were evidenced to enrich in HBT samples in comparison with their counterparts in HBPT samples (**Figure 3C**). According to our bulk RNA-seq data, we were curious about the major ferroptotic cells in HB tissues. To this aim, we computed the ferroptosis score with a set of characteristic genes for each particular cell populations (**Supplementary Table 2**). The highest ferroptosis scores were allocated to hepatoblasts, the major tumor cells, as well as macrophages, which possibly was a consequence or mechanism for immune suppression, as being proposed by many studies (31–34). The next tier of cells with strong ferroptosis included neutrophils and hepatocytes, at pretty much similar level (**Figure 3D**). Together, these data indicated that, in accordance with bulk RNA-seq result from sorted neutrophils, tissue neutrophils were endowed with strong ferroptosis signature.

### 3.4 Pseudo-time staging analysis mapped the dynamic features and ferroptosis fates of enriched terminally differentiated neutrophils in tumor

**Figure 4:**
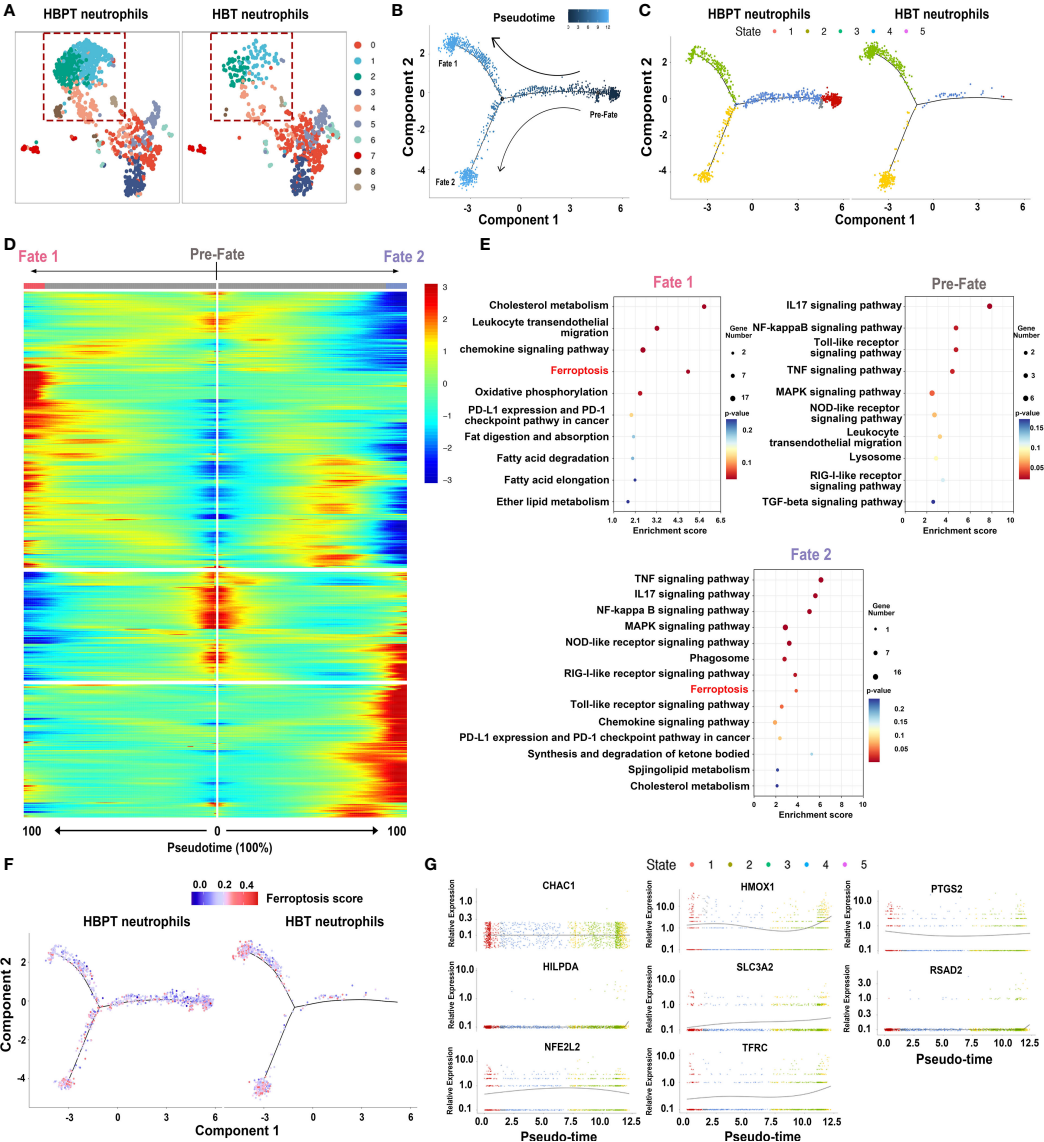
Dynamic features and ferroptosis fates of neutrophils by pseudo-time analysis. **(A)** UMAP plots of re-clustered neutrophils. **(B)** Monocle-guided cell trajectories of neutrophils colored from dark to light blue. The start of pseudo-time was indicated by dark blue and the end of pseudo-time by light blue, and the nodes represented branches of two differentiation trajectories, fate 1 and fate 2. **(C)** Monocle-guided cell trajectory of neutrophils colored by five different differentiation states. **(D)** Heatmap of differentially expressed genes, ordered based on their common kinetics through pseudo-time using Monocle. **(E)** Bubble chart showing the KEGG enrichment analysis of pre-fate, fate 1, and fate 2 neutrophils. **(F)** Monocle-guided cell trajectory of neutrophils colored by ferroptosis score. **(G)** Two-dimensional plots showing the dynamic expression of eight ferroptosis-related genes during the neutrophils aging state along the pseudo-time.

On the basis of the above ferroptosis analysis, we narrowed down our investigation on intratumoral neutrophils to further characterize their heterogeneity. To do this, we re-clustered neutrophils into 10 subclusters. Being illustrated in **Figure 4A**, neutrophils in both HBT and HBPT showed different gene expression profiles, whereas different ratios were noticed in some subclusters (dot line boxed). This implied that, regardless of the locations (tumor vs. para-tumor), neutrophils were heterogeneous subpopulations with DEGs. Taking the scRNA-seq of all denoted neutrophils, we performed pseudo-time analysis to see the development and differentiation stages. As shown in **Figure 4B**, neutrophils were divided into three development and differentiation fates with one at naïve stage (pre-fate) and two terminally differentiated stages with divergent cell fates (fate 1 and fate 2). Interestingly, when scRNA-seq data of neutrophils from para-tumor and tumor were analyzed separately, it became obvious that neutrophils in tumor exhibited increased maturity and were more terminally differentiated (**Figure 4C**). Comparison between neutrophils in pre-fate, fate 1, and fate 2 demarcated three discrete sets of gene enrichment elaborated on the fact that these neutrophils along different pseudo-time line were truly different functional subsets (**Figure 4D**). KEGG pathway analysis on these genes revealed, as our bulk RNA-seq indicated, that ferroptosis was one of the major functional characters in neutrophils (**Figure 4E**). Furthermore, when ferroptosis score was calculated, terminally differentiated neutrophils that were highly enriched in HB tumor were highly ferroptotic (**Figure 4F**). As supplement evidence, pseudo-time analysis for eight major genes responsible for ferroptosis revealed an overall trend of increasing expression along with the neutrophil maturation and differentiation (**Figure 4G**). It is important to point out that fate 2 neutrophils were clearly distinguishable from fate 1 in that this subset of neutrophils exhibited more immunosuppressive function in KEGG analysis such as PD-L1/PD1 immune checkpoint pathway, lipid, and fatty acid metabolism. In sum, compared with HBPT neutrophils, HBT neutrophils were predominantly well differentiated with high ferroptosis signature.

### 3.5 Ferroptotic intratumoral neutrophils suppressed anti-tumor function of T cells

**Figure 5:**
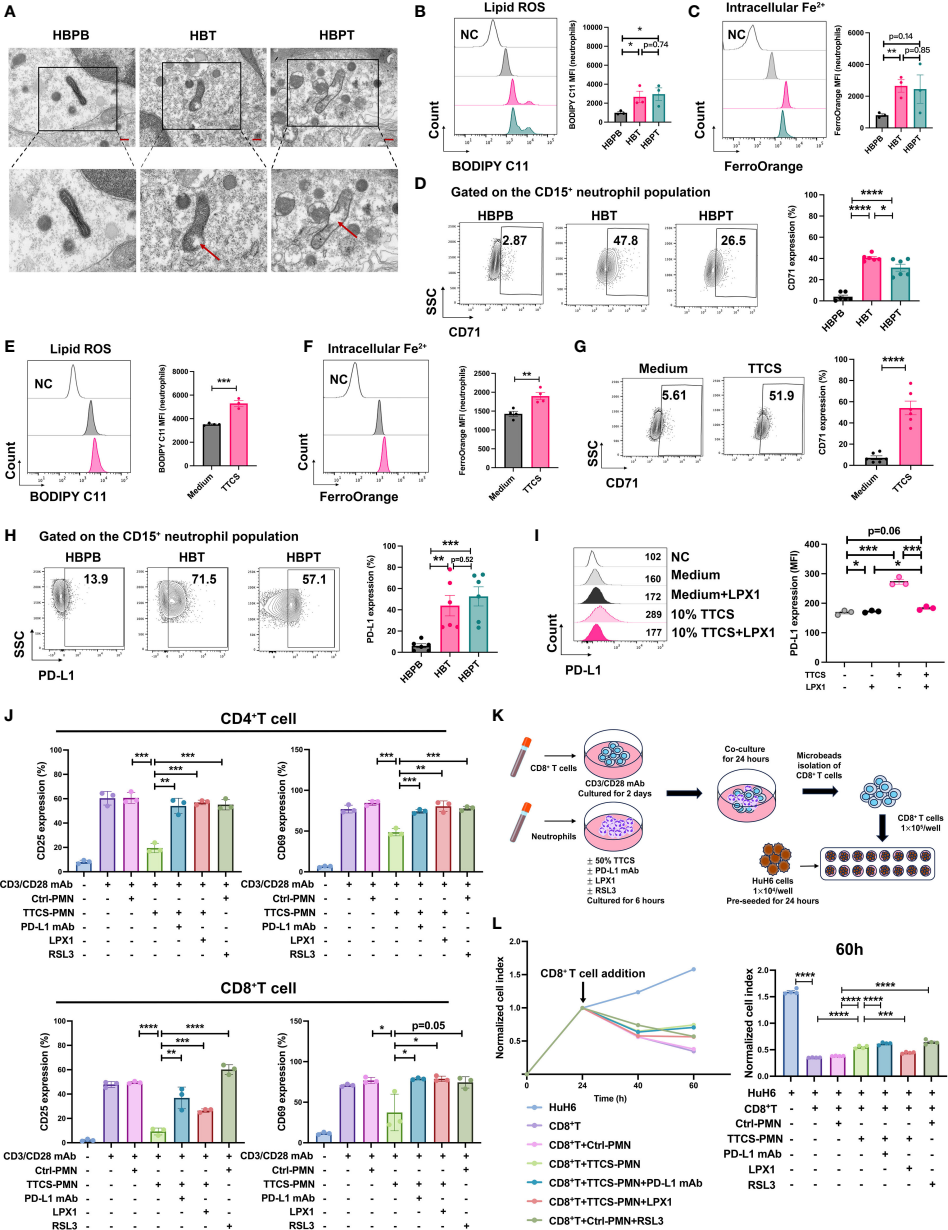
Enhanced ferroptosis and suppressive effects of neutrophils. **(A)** Representative images of transmission electron microscopy showed mitochondrial morphology changes in isolated neutrophils. The scale bar represents 2 μm (red). The lower panel showed a zoom on one mitochondrion in its corresponding image in the upper panel (black box). The red arrow indicated vacuole formation. **(B)** Flow analysis of BODIPY 581/591 C11-labeled lipoxidation of polyunsaturated fatty acids in isolated HBPB, HBT, and HBPT neutrophils (n = 3). MFI, mean fluorescence intensity. **(C)** FerroOrange probe was used to detect the intracellular Fe^2+^ of isolated HBPB, HBT, and HBPT neutrophils by flow cytometry (n = 3). **(D)** Expression of CD71 measured by flow cytometry (n = 6). **(E, F)** 50% TTCS-conditioned neutrophils were collected after 16 h for flow cytometric analysis of lipid ROS [**(E)** n = 3] and Fe^2+^ concentration [**(F)** n = 4], and CD71 expression [**(G)** n = 6], respectively. **(H, I)** Expression of PD-L1 on isolated neutrophils [**(H)** n = 6] or TTCS-treated neutrophils in the presence and absence of LPX1 [**(I)** n = 3]. **(J)** Flow cytometric analysis of CD4^+^ T-cell and CD8^+^ T-cell lymphocytes for expression of T-cell activation markers CD25 and CD69 after cocultured with 50% TTCS-conditioned neutrophils (n = 3). **(K)** Schematic illustration of the coculture steps for RTCA. **(L)** Cytotoxicity of CD8^+^ T-cell against HuH6 cells at an effector-to-target ratio of 10:1 was monitored by xCELLigence RTCA label-free technology. Arrows refer to the addition of CD8^+^ T cells. The y-axis is the normalized cell index generated by the RTCA software and displayed to reflect the vitality of tumor cells. Normalized cell index showed the average of four wells. Results for all measurements were mean ± SEM. *p < 0.05, **p < 0.01, ***p < 0.001, and ****p < 0.0001.

Experimentally, we further verified neutrophil ferroptotic features by transmission electron microscopy and found that a significant portion of neutrophils isolated from HBPB, HBT, and HBPT exhibited typical morphological changes in mitochondria such as decreased volume, vacuole formation, increased mitochondrial membrane density, as well as disappearance of mitochondrial cristae (**Figure 5A**). Along with this, lipid ROS level, both an indicator and a strong inducer of ferroptosis, was higher in tissue-infiltrated neutrophils (both tumor and para-tumor) than that of in blood (**Figure 5B**). FerroOrange staining and quantitative analysis by flow cytometry indicated increasing Fe^2+^ concentrations in tissue-infiltrated neutrophils (**Figure 5C**).

CD71, a known ferroptosis marker, was measured by flow cytometry, and we found that the percentage of CD71^+^ neutrophils was substantially higher in tumor tissue samples than that in blood samples. In addition, the fact that CD71^+^ neutrophils% was even higher in HB tumor tissue than in para-tumor tissue highlighted the single-cell sequencing result, suggesting an enriched ferroptotic terminally differentiated neutrophils in HB tumors (**Figure 5D**). To ensure that the ferroptosis of neutrophils is related to TME, isolated neutrophils from peripheral blood of HCs were treated with TTCS. Indeed, lipid ROS (**Figure 5E**) and intracellular Fe^2+^ (**Figure 5F**) and CD71 expression (**Figure 5G**) as well were successfully induced in neutrophils.

It is worth mentioning that, compared to blood neutrophils, the expression of PD-L1, one of essential immune checkpoint proteins, in tissue-infiltrated neutrophils is remarkably increased, suggesting an immunosuppressive feature of intratumoral neutrophils (**Figure 5H**). Similarly, PD-L1 could be readily induced on neutrophils by TTCS treatment. This induction of PD-L1 expression was related to neutrophils ferroptosis because adding ferroptosis inhibitor LPX1 in the culture abolished the expression induction (**Figure 5I**).

To validate the immunosuppressive role of neutrophils on T cells, we pre-treated microbeads isolated neutrophils with T cells and measured the T-cell activation by flow cytometry. As expected, TTCS-treated neutrophils dramatically subdued the activation of CD4^+^ and CD8^+^ T cells. Either PD-L1 neutralizing antibody or ferroptosis inhibitor LPX1 could restore the suppression effect of neutrophils against T cells. However, pre-treating neutrophils with a ferroptosis stimulant RSL3 was not able to affect T-cell activation, implying that functional transformation to neutrophils ferrroptosis is not the sole event to trigger the T-cell suppression (**Figure 5J**).

To investigate how suppressive neutrophils affect T-cell cytotoxicity to tumor cells, we designed experiment as diagrammed in **Figure 5K**. In brief, CD8^+^ T cells and neutrophils were sorted out. CD8^+^ T cells were stimulated with anti-CD3/28 for 2 days, and neutrophils were pretreated with various reagents respectively for 6 h. Then, CD8^+^ T cells were cocultured with pretreated neutrophils for another 24 h. CD8^+^ T cells then were sorted out to load into a 16-well plate pre-seeded with HuH6 tumor cells for cell killing assay (**Figure 5K**). As real-time cell analysis showed CD8^+^ T cells alone or CD8^+^ T cells pre-cultured with unconditioned neutrophils could effectively kill HuH6 tumor cells. The killing efficiency of CD8^+^ T cells was dampened by pre-coculture with TTCS-conditioned neutrophils implied that intratumoral neutrophils indoctrinated by TME might directly suppress infiltrated T-cell anti-tumor function. Unconditioned neutrophils, being pre-treated with ferroptosis inducer RSL3, were able to suppress tumoricidal activity of CD8^+^ T cells. Conversely, the inhibition of T-cell cytotoxicity by TTCS-conditioned neutrophils could be reversed by PD-L1 neutralizing antibody or ferroptosis inhibitor LPX1 (**Figure 5L**).

### 3.6 CXCL12-CXCR4 chemotaxis mediated neutrophil recruitment into tumor

**Figure 6:**
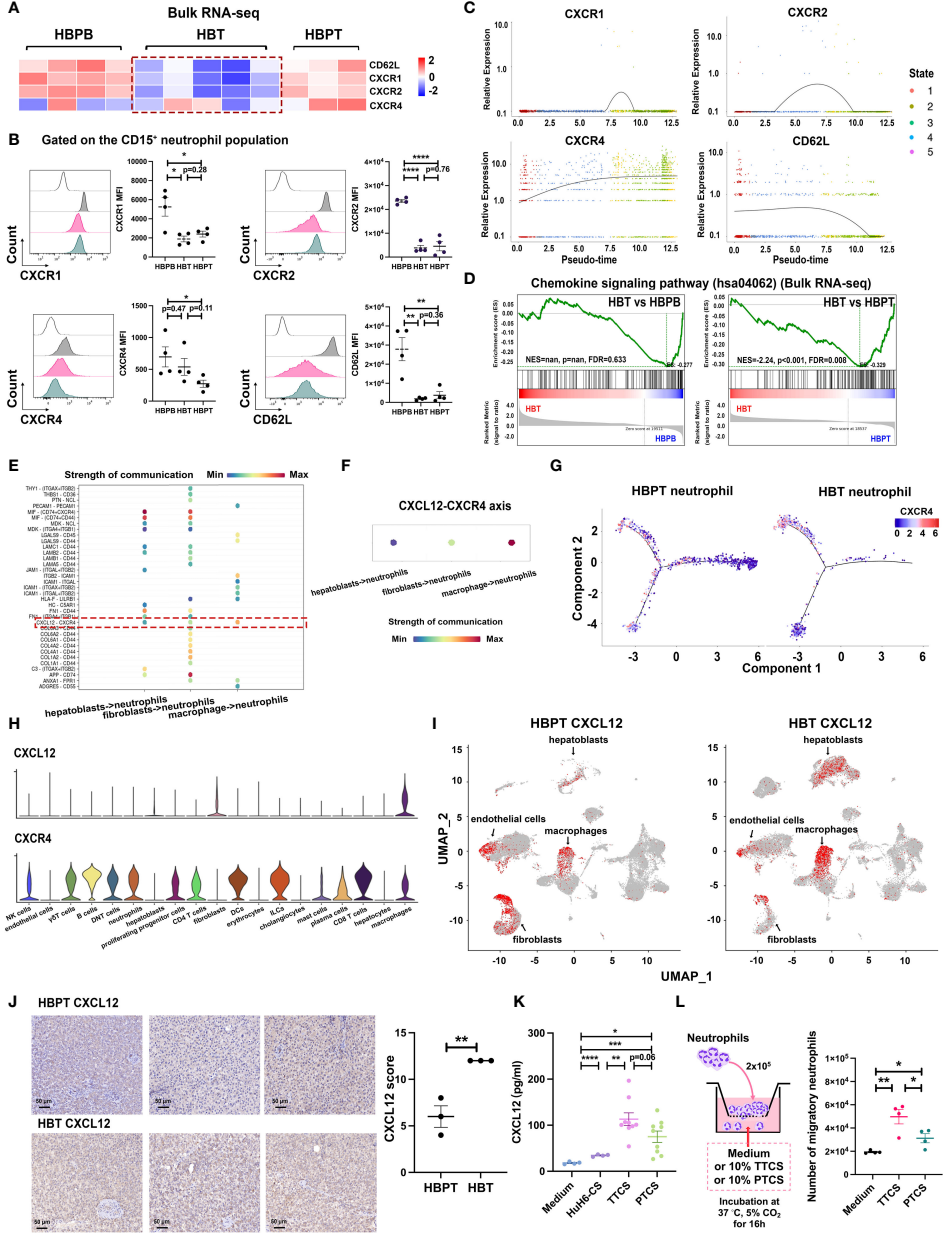
CXCL12-CXCR4 chemotaxis contributed to neutrophil recruitment. **(A)** Heatmap of four receptor-related gene expressions on isolated neutrophils using bulk RNA-seq transcriptome data. **(B)** Flow cytometric analysis of CXCR1, CXCR2, CXCR4, and CD62L expression gated on CD15^+^ neutrophil population (n = 4). **(C)** Two-dimensional plots showing the dynamic expression of CXCR1, CXCR2, CXCR4, and CD62L during the neutrophils aging state along the pseudo-time. **(D)** GSEA using bulk RNA-seq data from HBT versus HBPB and HBT versus HBPT shows enrichment of chemokine signaling pathway (hsa04062) in HBPT neutrophils. **(E)** Cellular interaction from other tissue-infiltrated cell types to neutrophils. **(F)** CXCL signaling pathway–related cellular interaction showed the activation of CXCL12-CXCR4 signaling to neutrophils. **(G)** Monocle-guided cell trajectory of neutrophils colored by CXCR4 expression. **(H)** Violin plots of CXCL12 and CXCR4 expression in all cell clusters. **(I)** UMAP-based distribution of CXCL12 in all cell clusters of HBPT and HBT. **(J)** Representative images (left) and evaluation (right) of immunohistochemistry staining of CXCL12 in HBT and HBPT, respectively. Scale bar, 50 μm. **(K)** Protein level CXCL12 in HuH6-CS (n = 4), TTCS (n = 9), and PTCS (n = 9) compared with medium control (n = 4) by ELISA. **(L)** Flow cytometry analysis of migratory neutrophils passing through the membrane (n = 4). Results for all measurements were mean ± SEM. *p < 0.05, **p < 0.01, ***p < 0.001, and ****p < 0.0001.

To further explore the relationship of neutrophils in tumor and their counterparts in para-tumor, we particularly focused on several well-characterized genes responsible for neutrophils chemotaxis including CXCR1, CXCR2, CXCR4, and CD62L. Our bulk RNA-seq data revealed that, compared with neutrophils from para-tumor tissues and blood, neutrophils sorted from tumor tissues expressed much lower level of CXCR1, CXCR2, and CD62L, but not CXCR4 (**Figure 6A**). As validated by our flow cytometry data, CXCR1, CXCR2, and CD62L were significantly downregulated in HBT neutrophils compared with that in HBPB neutrophils, whereas CXCR4 was still expressed (**Figure 6B**). We further analyzed the expression of them across pseudo-time staging analysis map. Along the trajectory, the expression of SELL was successionally decreased in pseudo-time, and the expression trends of CXCR1 and CXCR2 first increased and then decreased (**Figure 6C**). Note that, consistent with increased CXCR4 in HBT neutrophils analyzed by flow cytometry, CXCR4 expression was dramatically elevated across pseudo-time. Meanwhile, results from GSEA agreed that chemokine signaling pathway (hsa04062) was downregulated in HBT neutrophils compared with that in HBPT neutrophils (**Figure 6D**). Because neutrophil recruitment depends largely on tissue-specific chemokines (35, 36), we calculated the strength of cell-to-cell communication from perspective of chemotactic signaling pairs using scRNA-seq data. The calculated strength of communication illustrated in bubble plot indicated that, among 19 subset cell clusters, hepatoblasts, fibroblasts, and macrophages were the three cell populations interacted with neutrophils via CXCL12-CXCR4 chemotaxis (**Figures 6E, F**). Through pseudo-time trajectory analysis, expression of CXCR4 in neutrophils from tumor tissue was predominantly in fate 1 neutrophils, whereas, in para-tumor tissue, CXCR4 expression well dispersed in all maturation stages except for pre-fate naïve stage, which was enriched neutrophil population in para-tumor tissue. The focal expression of CXCR4 in fate 1 neutrophils in tumor tissue implied that terminally differentiated fate 1 neutrophils assumed to be immunosuppressive and ferroptotic could be the active immigrants taking CXCR4 as chemokines receptors (**Figure 6G**). When we interrogated cell origin of CXCR4 expression from scRNA-seq data, we found virtually all major immune cells such as neutrophils, macrophage, T and NK cells, and dendritic cells (DCs), but not non-immune cells such as endothelial cells, cholangiocytes, hepatoblasts, and hepatocytes expressed CXCR4. As for the ligand CXCL12, the expression was identified exclusively in macrophage, fibroblast, and hepatoblasts and minute amount in endothelial cells (**Figures 6H, I**). This fit very well to our cell-to-cell communication strength calculation as these cell types has the strongest interaction with neutrophils. Furthermore, in a CXCL12 immunohistochemistry staining comparison between tumor and para-tumor section, tumor tissue has signals greatly surpass the para-tumor tissue (**Figure 6J**). This difference in CXCL12 expression was secured by our ELISA experiment detecting CXCL12 in culture supernatant of HuH6 tumor cell line, isolated primary tumor cells from tumor region and from para-tumor region. While CXCL12 was barely detectable in HuH6 culture as a baseline, the concentration is significant higher in primary tumor cell culture than that in primary para-tumor cell culture (**Figure 6K**). Supporting this, in a Boyden chamber migration experiment, culture supernatant from primary tumor cells (TTCS) could induce more neutrophil migration events than culture supernatant from primary para-tumor cells (PTCS) (**Figure 6L**).

### 3.7 CXCR4^hi^ neutrophil subset exhibited higher ferroptosis tendency and activated immunosuppressive phenotype

**Figure 7:**
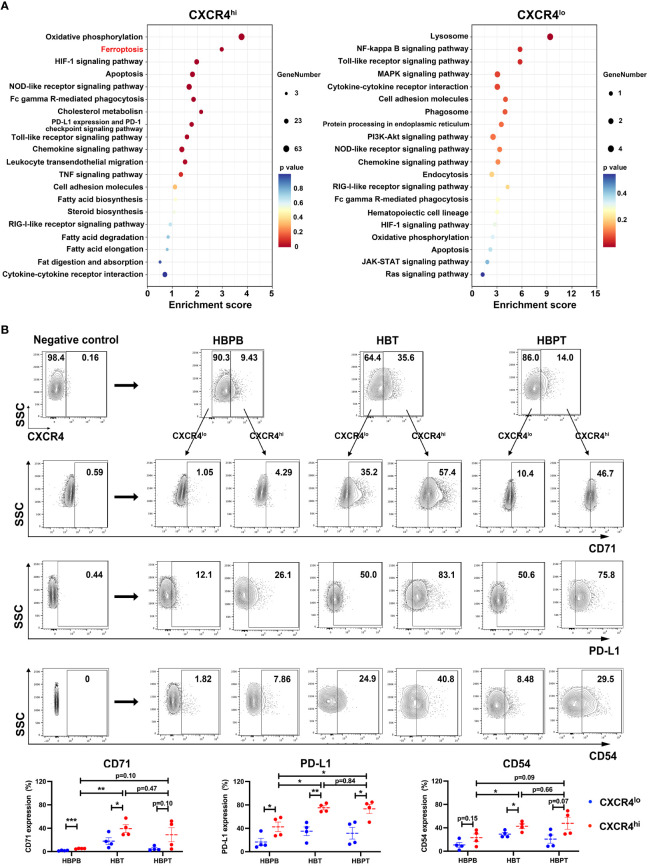
CXCR4^hi^ neutrophil subset with activated phenotype and ferroptosis tendency. **(A)** Bubble chart showing the KEGG enrichment analysis of the top 20 functional pathways between CXCR4^hi^ and CXCR4^lo^ neutrophil groups. **(B)** Flow cytometric assessment of CXCR4^hi^ and CXCR4^lo^ neutrophil proportions in HBPB, HBT, and HBPT, and compared the expression of CD71, PD-L1, and CD54 between CXCR4^hi^ and CXCR4^lo^ neutrophil (n = 4 per group). Results for all measurements were mean ± SEM. * p < 0.05, ** p < 0.01 and *** p < 0.001.

To substantiate the functional heterogeneity in CXCR4 expressing neutrophils, we divided all neutrophils into CXCR4^hi^ and CXCR4^lo^ neutrophils according to our scRNA-seq data and obtained DEGs between these two groups. Further KEGG analysis indicated that, as compared with CXCR4^lo^ neutrophils, ferroptosis and its related fatty acid metabolism pathways were the major functional characters of CXCR4^hi^ neutrophils, and this cohort of neutrophils also exhibited stronger immunosuppressive functions such as PD-L1/PD-1 immune checkpoint pathway (**Figure 7A**). Meanwhile, we compared the expression levels of CD71, PD-L1, and the activation marker CD54 between CXCR4^hi^ and CXCR4^lo^ neutrophil subsets from peripheral blood and tumor or para-tumor tissues experimentally. Our flow cytometry data demonstrated that, in contrast to CXCR4^lo^ neutrophils, CXCR4^hi^ neutrophil subset had evident ferroptotic, activated, and immunosuppressive signatures (**Figure 7B**).

**Figure 8:**
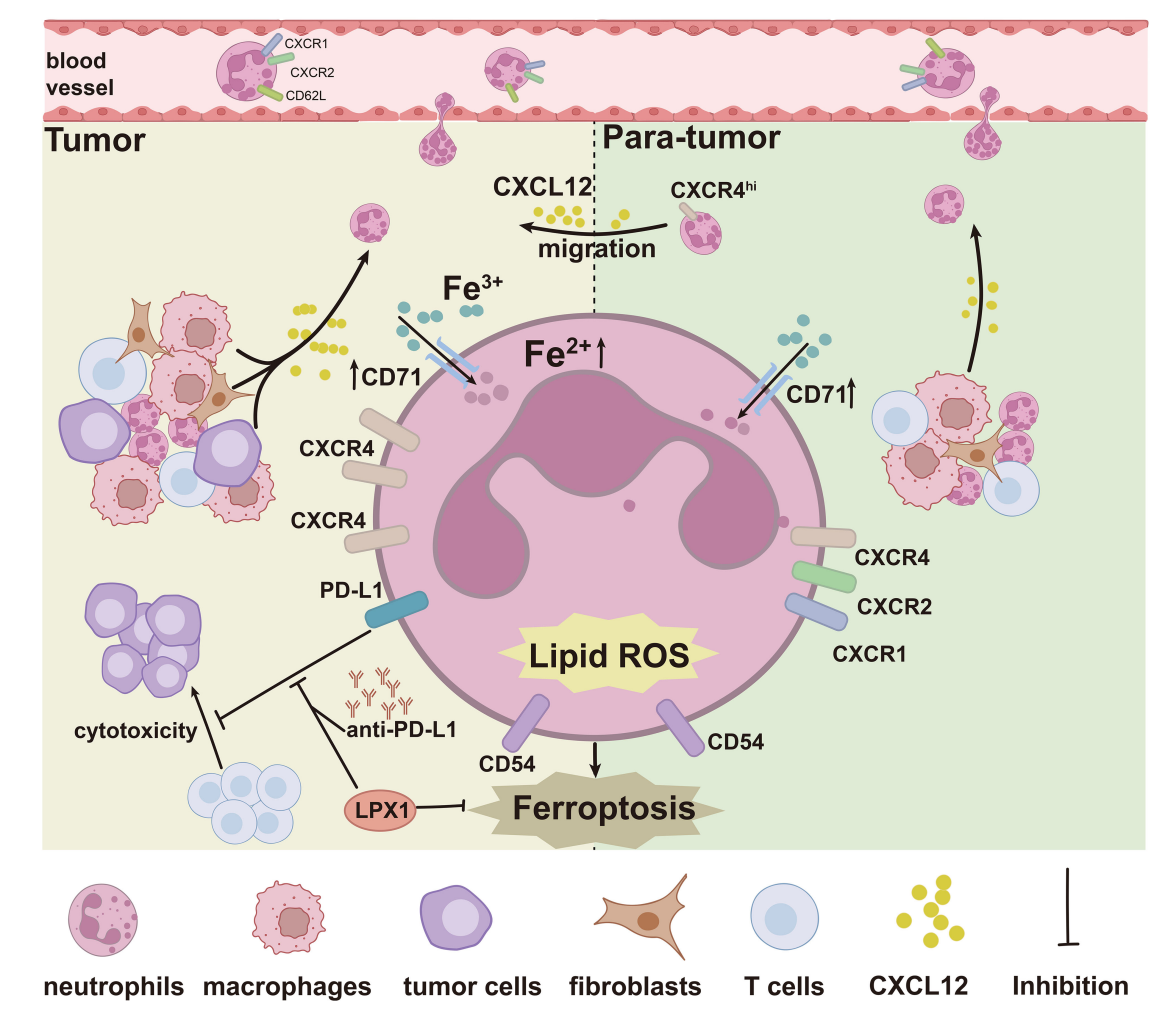
Graphic abstract. The graphic abstract clearly illustrated a significantly enhanced ferroptosis signature in HBT and HBPT tissue-infiltrated neutrophils with elevated intracellular Fe^2+^ concentration and lipid ROS generation. In addition to the tumor resident neutrophils, a cohort of CXCR4^hi^ neutrophils with activated phenotype, high ferroptosis tendency, and immunosuppressive molecule expression, migrating from HBPT, does exist in the tumor microenvironment.

These findings together implied that, in addition to the tumor resident neutrophils, there was also a cohort of CXCR4^hi^ neutrophils, with activated phenotype, high ferroptosis tendency and immunosuppressive molecule expression, migrating from HBPT (**Figure 8**).

## 4 Discussion

Neutrophils are immune cells with fascinating biology and functional plasticity. In our study, we used scRNA-seq techniques to provide evidence of distinct innate neutrophil subpopulations infiltrating the tumor in patients with HB, highlighting the heterogeneity and complexity of the immune landscape in HB. It was indicated that HB TME induced a ferroptotic and immunosuppressive phenotype in neutrophils accompanied by functional changes and surface protein expression, indicating that the presence of neutrophils in the complex landscape of the TME holds pivotal roles.

Immunotherapy has emerged as a promising strategy for the treatment of many cancers. It is unclear whether, and how, ferroptosis-prone neutrophils are involved in T-cell immunity and cancer immunotherapy. Neutrophils were reported to impact the success of immunotherapies, such as immune checkpoint blockade therapies, by displaying lymphocyte suppressive properties (37). Suppression of T cells by neutrophil via various mechanisms has been previously described (7, 38–40). A recent study demonstrated that spontaneous ferroptosis of neutrophils in the TME endowed them with immunosuppressive characteristics including influencing the activity of CD8^+^ T cells and tumor-associated macrophage (14). Notably, the expression of PD-L1 is a crucial mechanism in suppressing cytotoxic T lymphocyte function to induce immune tolerance and facilitate tumor escape from the immune system (41). Another study unveiled that xCT-mediated macrophage ferroptosis significantly increased PD-L1 expression in macrophages (42). In this study, we found that PD-L1 was highly expressed on HBT neutrophils, as described in other cancers (22). Unexpectedly, treatment of TTCS-conditioned neutrophils with ferroptosis inhibitor LPX1 resulted in substantial downregulation of PD-L1 expression. Moreover, blocking ferroptosis has been confirmed to improve the therapeutic efficacy of PD-L1/PD-1 blockade in glioblastoma (GBM) xenograft model, highlighting the important role of ferroptosis in tumor biology and clinical management and providing a novel synergic immunotherapeutic strategy that combines immune checkpoint blockade treatment with ferroptosis inhibition (43). This direct contact-mediated T-cell suppression was considered to be a dominant mechanism (44). Moreover, ferroptotic cells also exert contact-independent immune suppression by producing large amounts of PGE2 to increase the uptake and storage of lipids (14). Our results raise the question that whether the combination of ferroptosis inhibitor and anti–PD-L1/PD-1 treatment could reverse the pro-tumor polarity of neutrophils and restore the cytotoxic activity of T cells.

Our study confirmed upregulated ferroptosis in HB tissue-infiltrated neutrophils compared with circulating neutrophils. However, neutrophils in HBT and HBPT displayed absolutely different ferroptotic and chemotactic signatures. According to the pseudo-time staging analysis, HBPT neutrophils distributed dispersively in pre-fate and two terminal fate branches, whereas HBT neutrophils showed concentrated distribution in terminal fate branches. Both the two terminal fate neutrophils were featured with high ferroptotic scores, indicated by our bioinformatics analysis and experimental validation. Therefore, we wondered if a cohort of neutrophils infiltrated in tumors were migrated from para-tumors, contributing to the higher ferroptotic levels. In addition to the ferroptotic heterogeneity, changes in the chemokine signaling pathway were also significant in the HBT neutrophils. Neutrophils underwent increasing maturity from peripheral blood to HB para-tumor tissues and then to tumor. During this progression, CXCR1, CXCR2, and CD62L, essential for the neutrophil’s life cycle and regulating retention and release from the bone marrow (45–47), were downregulated, whereas CXCR4 became to be the only remaining chemokine receptor for recruiting neutrophils. HBT neutrophils at the terminal state tended to be mature cell characterized by a CXCR4^hi^CD62L^lo^ expression profile. Unexpectedly, chemotactic heterogeneity still exists between neutrophils from two terminal fate branches in tumor. Fate 2 neutrophils were featured with both innate immune functions, like pre-fate neutrophils and ferroptosis, but fate 1 neutrophils with higher expression of CXCR4 displayed ferroptotic and immunosuppressive signatures. Experimentally and consistently, CXCR4^hi^ neutrophils showed higher CD71 and PD-L1 expression, suggesting that fate 1 neutrophils were the culprit to undergo ferroptosis and suppress anti-tumor responses in HB tumor. With the high levels of ligand CXCL12 in HB tumor environment and the significance of CXCL12/CXCR4 axis for neutrophil release to liver (48), the surviving CXCR4 expression may result in this subset of ferrpototic neutrophil migrating into and then retenting in tumor. These findings together implied that, in addition to the resident neutrophils in HBT microenvironment, there was also a cohort of CXCR4^hi^ neutrophils that migrated from HBPT, with activated phenotype, higher ferroptosis tendency, and immunosuppressive molecule expression. Supportively, CXCR4^hi^CD62L^lo^ mature neutrophils have been shown to robustly promote tumor migration and support metastasis through the increased release of several metastasis-promoting factors, including NETs, ROS, vascular endothelial growth factor (VEGF) and matrix metalloproteinase-9 (MMP9). According to our RNA-seq results, NET-associated neutrophil elastase (NE), MMP9, PADI4, and HMGB1, were upregulated in HBT neutrophils, which were reported to awaken dormant cancer cells and facilitate cancer cell metastasis (49, 50).

To summarize, our study pinpointed that significantly enhanced ferroptosis signature in HBT and HBPT neutrophils, regardless of their differential distribution of cell maturation status, was the genuine perpetrators for immune suppression. Moreover, our data characterized a cohort of CXCR4^hi^ neutrophils, with activated phenotype, higher ferroptosis tendency, and increased immunosuppressive molecule expression, migrating from para-tumors via CXCL12/CXCR4 axis, suggesting a potential cell target for cancer immunotherapies.

## Data availability statement

The sequencing data that support the findings of this study have been deposited into CNGB Sequence Archive (CNSA) of China National GeneBank DataBase (CNGBdb) with accession number CNP0005379 (bulk RNA-seq) and CNP0005381 (scRNA-seq).

## Ethics statement

The studies involving humans were approved by Ethics Committee of Beijing Children’s Hospital. The studies were conducted in accordance with the local legislation and institutional requirements. Written informed consent for participation in this study was provided by the participants’ legal guardians/next of kin. Written informed consent was obtained from the individual(s) for the publication of any potentially identifiable images or data included in this article.

## Author contributions

ZL: Data curation, Formal Analysis, Investigation, Methodology, Project administration, Visualization, Writing – original draft. XW: Data curation, Formal Analysis, Software, Validation, Writing – review & editing, Project administration. JF: Methodology, Project administration, Resources, Writing – original draft. WC: Methodology, Project administration, Writing – original draft, Formal Analysis. WW: Investigation, Writing – original draft, Methodology. QW: Data curation, Methodology, Writing – original draft. SY: Resources, Writing – original draft, Methodology. WY: Methodology, Resources, Writing – original draft. YS: Methodology, Resources, Writing – original draft. WM: Data curation, Writing – original draft. YP: Methodology, Writing – original draft. HW: Investigation, Supervision, Writing – review & editing, Validation. JG: Funding acquisition, Investigation, Project administration, Validation, Visualization, Writing – review & editing, Supervision.
