# Supplementary material for: Intratumoral CXCR4hi neutrophils display ferroptotic and immunosuppressive signatures in hepatoblastoma
Source: Front Immunol. 2024 Feb 29;15:1363454. doi: 10.3389/fimmu.2024.1363454 (PMC10937446; doi:10.3389/fimmu.2024.1363454)
Supplement: Supplementary file 1 [file Table_1.docx]

Supplementary Table 1. Clinical characteristics of 18 children with hepatoblastoma.

| No. | Sex | Age (months) | Symptom | AFP (ng/ml) | Maximum diameter of tumor (cm) | PRE-TEXT staging | Metastases | Pathology | Risk stratification of CHIC | Follow-up (months) | Prognosis |
| --- | --- | --- | --- | --- | --- | --- | --- | --- | --- | --- | --- |
| P1 | M | 43 | abdominal distension | 134552 | 14.2 | IV | no | fetal + embryonal + mesenchymal | high | 11 | alive |
| P2 | M | 8 | abdominal distension | 625540 | 12.9 | III | no | fetal + embryonal + mesenchymal | low | 8 | alive |
| P3 | F | 46 | abdominal mass | 10716 | 13.4 | II | no | fetal + embryonal | low | 7 | alive |
| P4 | M | 24 | abdominal mass | 15933 | 11.9 | II | no | fetal + embryonal | low | 8 | alive |
| P5 | M | 21 | abdominal mass | 1363.77 | 8.2 | II | no | fetal + embryonal | low | 14 | alive |
| P6 | F | 16 | abdominal mass | 1016759 | 14 | II | no | fetal + embryonal + mesenchymal | intermediate | 10 | alive |
| P7 | F | 27 | abdominal mass | 517281 | 16.0 | III | no | fetal + embryonal | high | 16 | alive |
| P8 | M | 34 | fever | 382074 | 11.6 | II | lung | fetal + embryonal | high | 14 | alive |
| P9 | M | 8 | abdominal mass | 23339 | 5.9 | II | no | fetal + embryonal | low | 9 | alive |
| P10 | F | 59 | vomit | 405.94 | 6.8 | II | no | fetal | very low | 6 | alive |
| P11 | M | 38 | abdominal mass | 1026 | 12.6 | II | no | fetal + embryonal | very low | 1 | alive |
| P12 | M | 29 | abdominal mass | 316238 | 9.7 | IV | lung | fetal + embryonal + mesenchymal | high | 11 | alive |
| P13 | M | 41 | fever | 215164 | 11.6 | I | lung | fetal + embryonal + mesenchymal | high | 9 | alive |
| P14 | F | 27 | abdominal mass | 1598530 | 13.7 | IV | no | fetal + embryonal + mesenchymal | intermediate | 5 | alive |
| P15 | F | 31 | abdominal mass | 753956 | 12.5 | III | no | fetal + embryonal + mesenchymal | intermediate | 4 | alive |
| P16 | M | 79 | abdominal mass | 521892 | 10.5 | III | lung | fetal + embryonal | high | 4 | alive |
| P17 | F | 59 | abdominal mass | 6830 | 12.1 | III | no | fetal + embryonal | intermediate | 2 | alive |
| P18 | F | 9 | abdominal distension | 688920 | 6.7 | II | no | fetal + embryonal + mesenchymal | intermediate | 1 | alive |

AFP, Alpha-Fetoprotein; PRE-TEXT, PRE-treatment EXTent of tumor; CHIC, Children's Hepatic Tumor International Collaboration
