# Supplementary material for: Intratumoral CXCR4hi neutrophils display ferroptotic and immunosuppressive signatures in hepatoblastoma
Source: Front Immunol. 2024 Feb 29;15:1363454. doi: 10.3389/fimmu.2024.1363454 (PMC10937446; doi:10.3389/fimmu.2024.1363454)
Supplement: Supplementary file 2 [file Table_2.docx]

Supplementary table 2 Genes applied to calculate the ferroptosis score

| Gene | Description |
| --- | --- |
| SLC40A1 | Solute Carrier Family 40 Member 1 |
| NCOA4 | Nuclear Receptor Coactivator 4 |
| FANCD2 | FA Complementation Group D2 |
| PNPLA2 | Patatin Like Phospholipase Domain Containing 2 |
| LPCAT3 | Lysophosphatidylcholine Acyltransferase 3 |
| CARS | Calcium Sensing Receptor |
| BAP1 | BRCA1 Associated Protein 1 |
| PCBP2 | Poly (RC) Binding Protein 2 |
| GCLM | Glutamate-Cysteine Ligase Modifier Subunit |
| CISD1 | CDGSH Iron Sulfur Domain 1 |
| SLC7A11 | Solute Carrier Family 7 Member 11 |
| RPL8 | Ribosomal Protein L8 |
| CRYAB | Crystallin Alpha B |
| TF | Tissue Factor |
| CHAC1 | ChaC Glutathione Specific Gamma-Glutamylcyclotransferase 1 |
| CP | Ceruloplasmin |
| HILDPA | Hypoxia Inducible Lipid Droplet Associated |
| HSPB1 | Heat Shock Protein Family B (Small) Member 1 |
| AKR1C1 | Aldo-Keto Reductase Family 1 Member C1 |
| TFRC | Transferrin Receptor |
| PRNP | Prion Protein (Kanno Blood Group) |
| GSS | Glutathione Synthetase |
| GPX4 | Glutathione Peroxidase 4 |
| ALOX15 | Arachidonate 15-Lipoxygenase |
| STEAP3 | STEAP3 Metalloreductase |
| DPP4 | Dipeptidyl Peptidase 4 |
| HMGCR | 3-Hydroxy-3-Methylglutaryl-CoA Reductase |
| GLS2 | Glutaminase 2 |
| VDAC2 | Voltage Dependent Anion Channel 2 |
| ACSL4 | Acyl-CoA Synthetase Long Chain Family Member 4 |
| EMC2 | ER Membrane Protein Complex Subunit 2 |
| HMOX1 | Heme Oxygenase 1 |
| ATP5G3 | ATP Synthase Membrane Subunit C Locus 3 |
| VDAC3 | Voltage Dependent Anion Channel 3 |
| PCBP1 | Poly (RC) Binding Protein 1 |
| CS | Citrate Synthase |
| TP5 | Thymopoietin 5 |
| HIF1A | Hypoxia Inducible Factor 1 Subunit Alpha |
| ATG5 | Autophagy Related 5 |
| ATG7 | Autophagy Related 7 |
| SAT1 | Spermidine/Spermine N1-Acetyltransferase 1 |
| FTH1 | Ferritin Heavy Chain 1 |
| ABCA1 | ATP Binding Cassette Subfamily A Member 1 |
| SLC3A2 | Solute Carrier Family 3 Member 2 |
| MT1G | Metallothionein 1G |
| CD44 | CD44 Molecule (Indian Blood Group) |
| PTGS2 | Prostaglandin-Endoperoxide Synthase 2 |
| NFE2L2 | NFE2 Like BZIP Transcription Factor 2 |
| FDFT1 | Farnesyl-Diphosphate Farnesyltransferase 1 |
