# Supplementary material for: Intratumoral CXCR4hi neutrophils display ferroptotic and immunosuppressive signatures in hepatoblastoma
Source: Front Immunol. 2024 Feb 29;15:1363454. doi: 10.3389/fimmu.2024.1363454 (PMC10937446; doi:10.3389/fimmu.2024.1363454)
Supplement: Supplementary file 3 [file DataSheet_1.docx]

## **Supplementary Figure Legends**

**
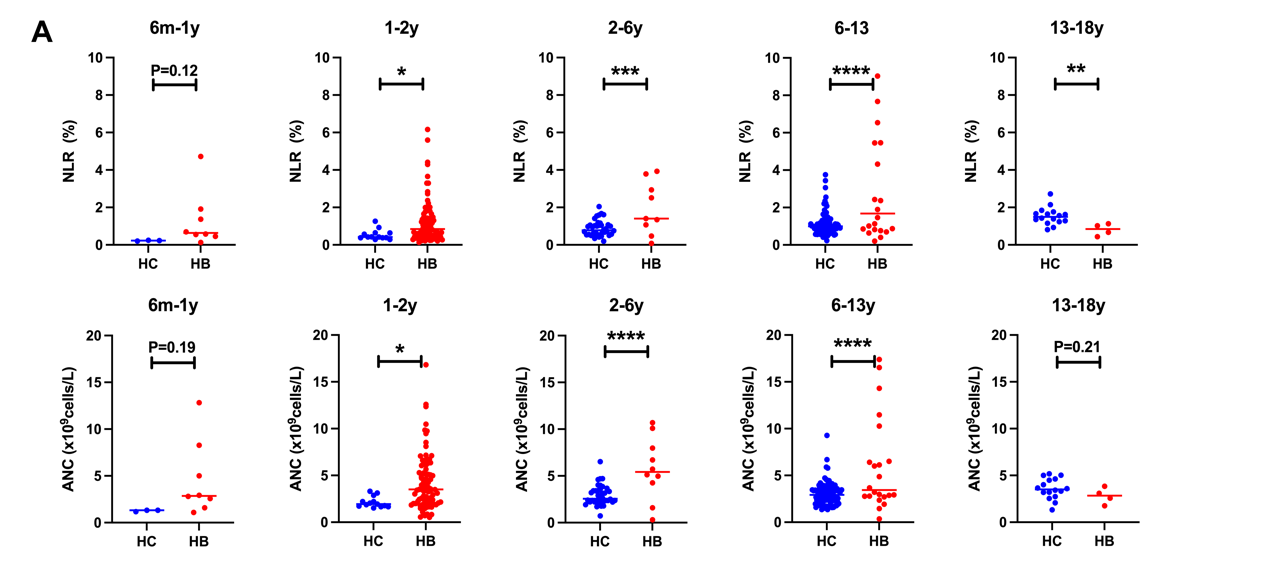
**

**Supplementary Figure 1. NLR and ANC analysis in age groups**

Comparison of NLR and ANC in five age groups between HC (n = 160) and HB patients (n = 127).

**
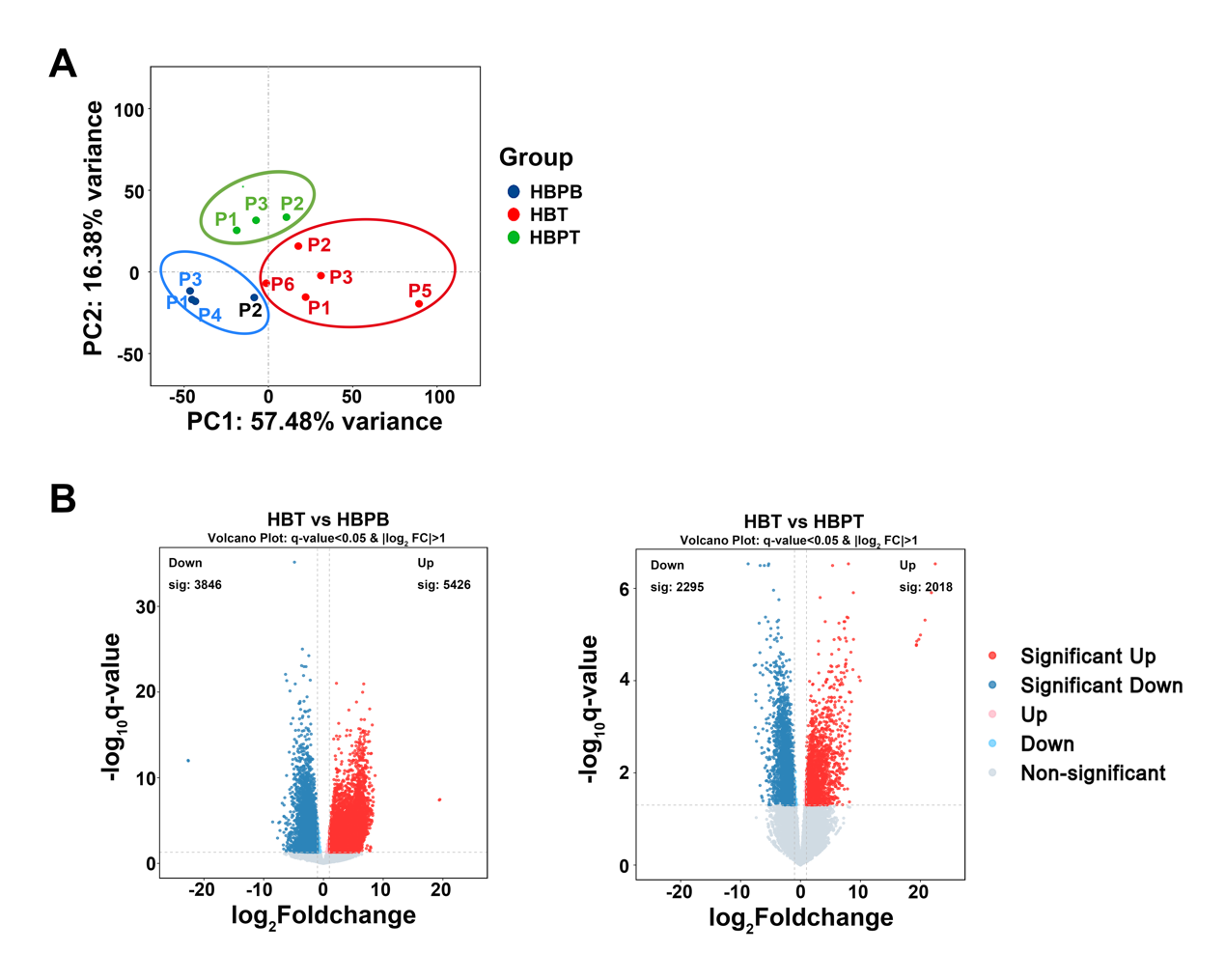
**

**Supplementary Figure 2. NLR and ANC analysis in age groups**

(A) Principal component analysis (PCA) was performed using the 1,000 most variable genes from neutrophils from individuals with HBPB (n = 4), HBT (n = 5) and HBPT (n = 3). (B) Volcano plots depicting DEG analysis between HBT vs. HBPB neutrophils (left), HBT vs. HBPT neutrophils (right). Blue and red marked genes with *q* value < 0.05 & log_2_ (fold-change) < -1 and *q* value < 0.05 & log_2_ (fold-change) >1, respectively.

**
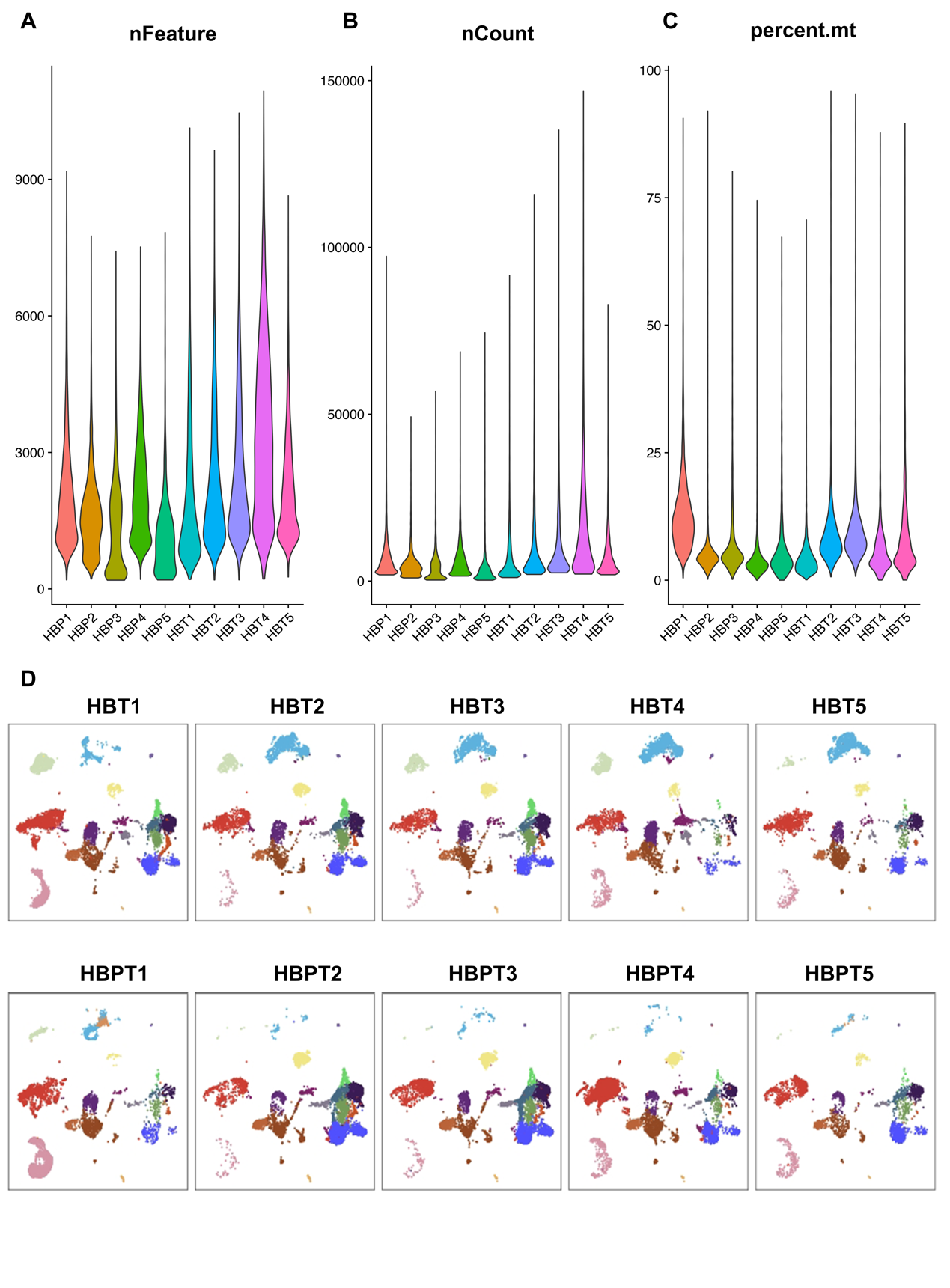
**

**Supplementary Figure 3. Quality control of single-cell RNA sequencing data**

(A) Violin plots depicting the number of genes detected in each sample (nFeature). (B) Violin plots depicting the number of unique RNA molecules detected in each sample (nCount). (C) Plots depicting the percentage of reads mapping to the mitochondrial genome (percent.mt). (D) Classification of cell clusters in each sample.


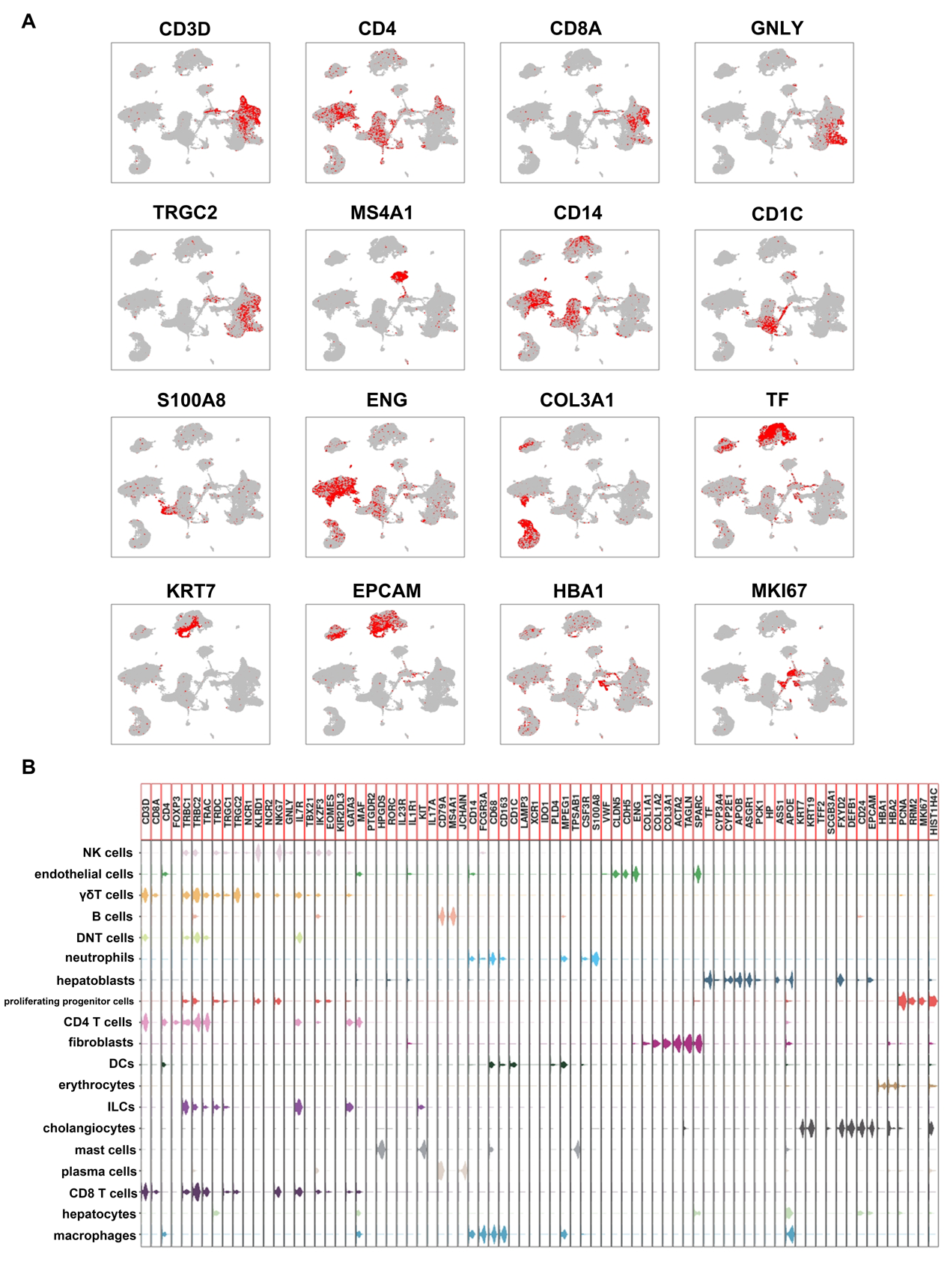


**Supplementary Figure 4. Marker genes for cell type clustering based on the single cell translatome**

(A) UMAP plots showing the distribution of different cell clusters. (B) Violin plots showing the expression level of representative markers across different subtypes.
